# Medical Changes and Appointments

**Published:** 1847-09

**Authors:** 


### MEDICAL CHANGES AND APPOINTMENTS.

Prof. John P. Harrison has been transferred to the chair of Theory and Practice of Medicine in the Medical College of Ohio. He is succeeded in the chair of Materia Medica by Prof. L. M. Lawson, late of the Medical Department of Transylvania University. Prof. Dickson of the Medical College at Charleston, S. C., has been selected to fill the vacancy occasioned by the death of Prof. Revere, in the Medical Department of the New York University. Prof. Henry, of Princeton, takes the Chemical chair vacated by Dr. Hare in the Pennsylvania University.

